# Integrated Genomic Analysis Uncovers the Evolutionary Landscape and Global Dissemination of Senecavirus A

**DOI:** 10.3390/vetsci13050429

**Published:** 2026-04-28

**Authors:** Wenqiang Wang, Suhao Zhang, Qilin Zhao, Liping Jiang, Zhenbang Zhu, Wei Wen, Xiangdong Li

**Affiliations:** 1 Jiangsu Co-Innovation Center for Prevention and Control of Important Animal Infectious Diseases and Zoonoses, College of Veterinary Medicine, Yangzhou University, Yangzhou 225009, China; wqwang@yzu.edu.cn (W.W.); mz120241796@stu.yzu.edu.cn (S.Z.); mx120241033@stu.yzu.edu.cn (Q.Z.); mx120241036@stu.yzu.edu.cn (L.J.); 007583@yzu.edu.cn (Z.Z.); wenw@yzu.edu.cn (W.W.); 2 Jiangsu Interdisciplinary Center for Zoonoses and Biosafety, Yangzhou University, Yangzhou 225009, China; 3 Jiangsu Key Laboratory of Zoonosis, Yangzhou University, Yangzhou 225009, China; 4 Joint International Research Laboratory of Agriculture and Agri-Product Safety, The Ministry of Education of China, Yangzhou University, Yangzhou 225009, China

**Keywords:** Senecavirus A evolution, phylogenetics, recombination, haplotype, purifying selection, phylogeography

## Abstract

Senecavirus A (SVA) has rapidly emerged as a globally distributed swine pathogen, with clinical signs mimicking vesicular diseases such as Foot-and-Mouth Disease, posing challenges for timely detection and control. Here, we analyzed 329 complete SVA genomes spanning multiple continents to provide a comprehensive view of its evolutionary dynamics, recombination patterns, haplotype diversity, and global dissemination. Phylogenetic analyses revealed two major lineages: Lineage 1, consisting mainly of early strains from the United States before 2007, and Lineage 2, which emerged post-2007 and subsequently spread across the Americas and East Asia. Recombination was confined to Lineage 2 and concentrated in nonstructural regions, particularly 2C, highlighting intra-lineage genetic exchange as a driver of recent diversification. Haplotype analysis of the 3AB gene identified 170 distinct haplotypes, revealing a star-like network structure consistent with rapid population expansion from a central ancestral variant, while secondary branches reflect ongoing regional diversification. Despite this high genetic variation, genome-wide dN/dS ratios remained below one, and purifying selection was strongest in the N-terminal domains of structural and nonstructural proteins, indicating functional constraints that maintain viral fitness. Time-scaled phylogenetic reconstruction and Bayesian Skyline analysis revealed rapid lineage diversification and a marked increase in effective population size in the early 2010s. Phylogeographic inference further identified repeated introductions from the Americas into East Asia, likely facilitated by swine trade and other anthropogenic factors. Collectively, SVA evolution is driven by frequent mutation and intra-lineage recombination yet constrained by pervasive purifying selection, generating extensive genetic diversity while maintaining functional integrity, with implications for genomic surveillance and targeted control.

## Simple Summary

Senecavirus A (SVA) is an emerging virus affecting pigs worldwide and can cause clinical signs similar to serious vesicular diseases, making it difficult to diagnose in the field. In this study, we analyzed a large collection of viral genomes from different regions to better understand how this virus evolves and spreads. We found that the virus has become more widespread in recent years and continues to diversify genetically, partly through mutation and recombination. However, important viral functions remain conserved, suggesting evolutionary constraints. Our results also indicate that international animal movement may contribute to its spread. These findings provide useful information for improving surveillance and developing more effective control strategies.

## 1. Introduction

Senecavirus A (SVA) is an emerging swine pathogen that has garnered increasing attention over the past two decades due to its rapid global spread and economic impact on the pig industry [1]. SVA is a non-enveloped, positive-sense single-stranded RNA virus belonging to the genus *Senecavirus* within the family *Picornaviridae* [2], which also includes other economically significant viruses such as Foot-and-Mouth Disease Virus and Swine Vesicular Disease Virus [3]. Clinically, SVA infection induces vesicular lesions in the oral cavity, hooves, and interdigital spaces, often indistinguishable from notifiable vesicular diseases [4], complicating differential diagnosis and timely outbreak response. Although pigs are the primary host [5], SVA RNA has also been detected in non-swine species [6,7], suggesting a broader host range and raising concerns about potential reservoirs that could contribute to virus persistence and spread.

Since the first reports of SVA in the United States in 2007 [8], outbreaks have been increasingly documented across the Americas and Asia, with periodic emergence in previously unaffected regions [9,10,11]. Despite its growing epidemiological importance, fundamental questions regarding SVA evolution and global dissemination remain unresolved [12]. Its evolutionary origin and potential ancestral host range also remain unclear, and historical cross-species transmission events cannot be excluded, although it is phylogenetically placed within the Picornaviridae family and primarily associated with swine. Existing studies have largely focused on regional outbreaks or small datasets, providing limited insight into the long-term evolutionary dynamics, lineage diversification, and patterns of intercontinental transmission [13]. In particular, the mechanisms generating genetic diversity remain unclear at the global scale: the relative contributions of mutation, recombination, and natural selection have not been systematically characterized. Moreover, unlike many RNA viruses that often evolve under strong positive selection [14,15,16,17], it remains unclear how SVA generates genetic diversity while maintaining functional integrity of key viral proteins. Understanding the potential balance between mutational input and functional constraint is critical for elucidating the evolutionary mechanisms that underpin SVA adaptability and persistence.

To address these knowledge gaps, we conducted a comprehensive analysis of all publicly available complete SVA genomes, integrating phylogenetic reconstruction, recombination detection, haplotype network analysis, codon-level selection pressure estimation, and phylogeographic modeling. By combining these complementary approaches, our study establishes a unified framework for understanding SVA evolution and global spread. The resulting insights illuminate how a virus with high mutational potential can maintain functional integrity, adapt to diverse ecological contexts, and traverse international boundaries via complex trade and movement networks. This integrated perspective not only fills critical gaps in our understanding of SVA but also provides a foundation for evidence-based molecular surveillance, risk assessment, and the development of targeted intervention strategies. Ultimately, elucidating the evolutionary and dissemination dynamics of SVA is essential for mitigating its impact on swine health and safeguarding international trade in pork products.

## 2. Materials and Methods

### 2.1. Sequence Dataset and Alignment

All available complete SVA genome sequences (*n* = 329), with sampling dates ranging from 1988 to 2025, were downloaded from the GenBank database of the National Center for Biotechnology Information (NCBI) (https://www.ncbi.nlm.nih.gov/), accessed on 28 October 2025. Metadata associated with each sequence, including sampling date, geographic origin, and strain identifier, were also collected. Multiple sequence alignment of all genomes was performed using the L-INS-i algorithm implemented in MAFFT (version 7.518) [18]. The resulting alignments was inspected and manually adjusted to ensure correct codon alignment. Ambiguously aligned regions were removed where necessary. Reading frames and overall alignment quality were subsequently verified.

### 2.2. Phylogenetic Analysis

Maximum-likelihood phylogenetic trees were inferred from the complete SVA genome alignment using IQ-TREE (version 2.2.0) [19]. Nodal support was assessed with 1000 ultrafast bootstrap replicates. The best-fitting nucleotide substitution model (GTR+F+R3) was selected based on the Bayesian Information Criterion (BIC). Phylogenetic trees were visualized using FigTree (version 1.4.4) and subsequently annotated and refined for presentation using iTOL (https://itol.embl.de/) [20].

### 2.3. Recombination Analysis

Potential recombination events within the aligned complete SVA genomes were screened using RDP4 (version 4.101) [21]. Seven recombination detection methods implemented in the package (RDP, GENECONV, Chimaera, MaxChi, BootScan, 3Seq, and SiScan) were applied. To reduce false positives, only recombination events supported by at least four methods with a significance threshold of *p* < 0.05 were considered credible. These putative events were further examined using SimPlot (version 3.5.1) [22], where similarity plot and bootscan analyses were performed to verify recombination signals and refine breakpoint positions. In addition, phylogenetic network analysis was conducted using SplitsTree (version 6.3.30) to visualize conflicting phylogenetic signals associated with recombination [23]. Recombination plots and related visualizations were generated using R (version 4.5.2).

### 2.4. Haplotype Analysis

Sequence conservation and haplotype characteristics of SVA were initially assessed using DnaSP6 (version 6.12.03) [24]. Key parameters, including the number of polymorphic sites (S), haplotype (gene) diversity (Hd), and nucleotide diversity (π), were calculated. Based on these data, a Nexus file was generated for haplotype network construction. Haplotype networks were constructed using POPART (version 1.7) based on the median-joining network algorithm. The networks were visualized to depict relationships among haplotypes and their relative frequencies.

### 2.5. Selection Pressure Analysis

To reduce computational burden, 18 SVA strains representing major lineages and sampling regions were selected for selection analysis. Gene-level selection was assessed using PAML (version 4.9j) to estimate the nonsynonymous/synonymous substitution rate ratio (*ω*) for different coding regions [25], where *ω* < 1 indicates purifying selection, *ω* = 1 neutral evolution, and *ω* > 1 positive selection. To identify individual codons under selection, analyses were performed on the Datamonkey web server using three complementary methods: FEL [26], MEME [27], and FUBAR [28]. Only codon sites inferred to be under purifying (negative) selection by at least two complementary methods were retained for downstream interpretation.

### 2.6. BEAST Analysis

Aligned SVA genome sequences were first evaluated for temporal signal using root-to-tip regression implemented in TempEst (version 1.5.3) [29]. Sequences showing clear deviation from the overall regression pattern, indicating inconsistent temporal signal, were identified as outliers and excluded prior to downstream molecular clock analyses to improve the reliability of time-scaled inference. Time-scaled phylogenetic inference was then performed in BEAST (version 1.10.4) [30], with XML configuration files generated using BEAUti (version 1.10.4). The nucleotide substitution model was selected using ModelFinder (version 2.2.0) implemented in IQ-TREE 2 based on Bayesian Information Criterion scores, and the best-fitting GTR+Γ4 model was applied in subsequent analyses [19]. All codon positions were unpartitioned, substitution model parameters were linked across the alignment.

Both strict and relaxed molecular clock models, as well as alternative coalescent tree priors, were evaluated. The final configuration employed a strict molecular clock and a coalescent Bayesian Skyline prior, which provided stable convergence and consistent posterior estimates. Markov chain Monte Carlo (MCMC) analyses were run for 200 million generations, sampling every 20,000 steps, with the initial 10% of samples discarded as burn-in [31]. Convergence and mixing were assessed in Tracer v1.7, with all parameters exhibiting effective sample sizes (ESS) greater than 200 [32]. The maximum clade credibility (MCC) tree was summarized using TreeAnnotator (version 1.10.4), with node heights summarized as median posterior estimates [33].

Discrete phylogeographic reconstruction was conducted using Bayesian stochastic search variable selection (BSSVS) to identify statistically supported migration pathways. Calculation of Bayes factors and posterior probabilities, as well as visualization of transmission pathways, was performed using SpreaD3 v0.9.7 [34]. Only diffusion routes with Bayes factors (BF) > 3 and posterior probabilities (PP) > 0.5 were retained for interpretation. The inferred migration network was visualized using R.

## 3. Results

### 3.1. Temporal and Geographic Divergence of the Two SVA Lineages

**Figure 1:**
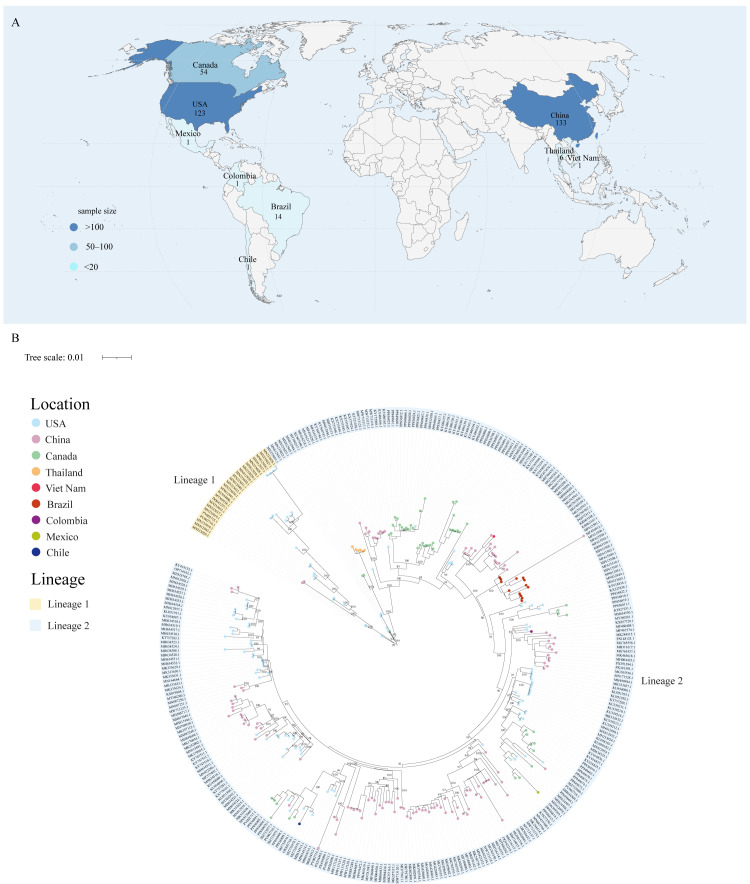
Global sampling distribution and phylogenetic structure of SVA. (**A**) Geographic distribution and sampling density of SVA sequences. Darker shading represents countries with more submitted sequences and therefore greater sampling density. (**B**) Maximum-likelihood phylogeny of complete SVA genomes. Outer colored ranges indicate viral lineages, while inner colored nodes represent the countries of origin.

Among the 329 complete SVA genome sequences retrieved from NCBI, the majority originated from China (*n* = 129) and the United States (*n* = 122), followed by Canada (*n* = 54), Brazil (*n* = 14), and Thailand (*n* = 6), while Mexico, Colombia, Viet Nam, and Chile each contributed a single sequence (Figure 1A).

Phylogenetic analysis revealed two well-supported lineages (bootstrap support = 99%) with distinct temporal and geographic distributions (Figure 1B). Lineage 1 consists predominantly of sequences collected in the United States before 2007, including the reference strain SVV-001. These strains exhibit strong geographic clustering, indicating that early SVA transmission was largely confined to the United States. In contrast, Lineage 2 strains emerged predominantly after 2007 and have since circulated widely across the Americas and Asia, demonstrating a pattern of extensive transboundary spread. Notably, based on genetic distance, three sequences collected in the United States before 2007 (MN233022.1, MN812943.1, and MN233021.1) cluster within Lineage 2 and were thus assigned to it.

### 3.2. Recombination Drives Genetic Diversification in Recent SVA Outbreaks

**Figure 2:**
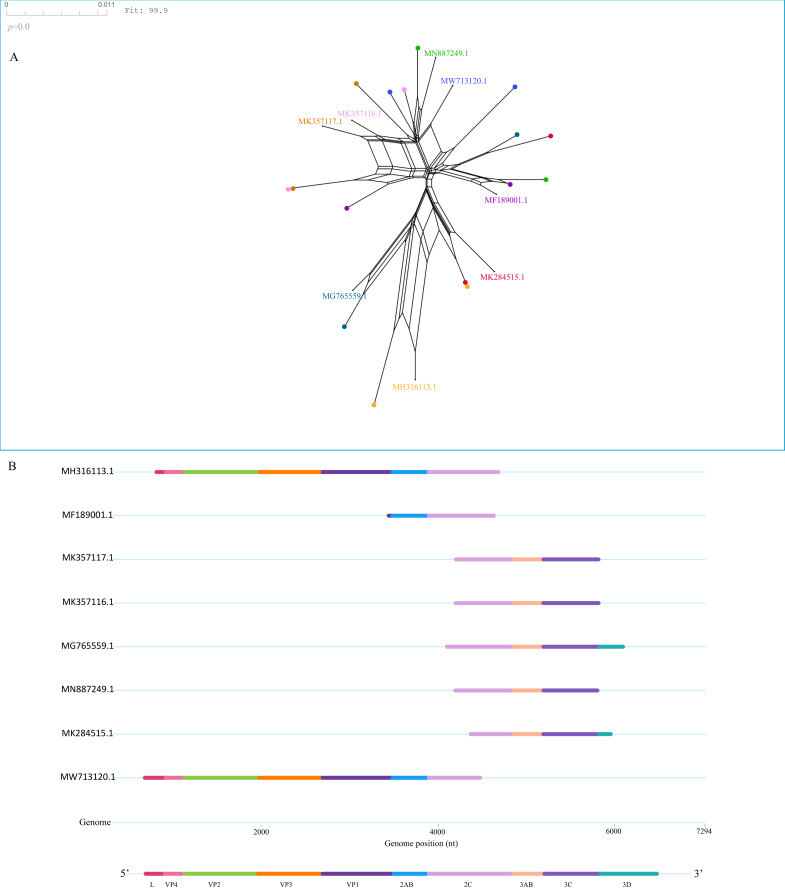
SplitsTree network and genome-wide mapping of recombination. (**A**) Phylogenetic network analysis reveals recombination events among SVA strains. Recombinant strains are indicated by their GenBank accession numbers, and nodes correspond to putative parental sequences. Intersecting boxes in the network represent conflicting phylogenetic signals, indicating potential recombination events among these sequences. (**B**) Genome-wide recombination patterns of SVA. Each horizontal bar corresponds to a complete genome, and colored segments indicate regions inferred to be derived from different parental lineages. Genome coordinates (nt) are shown along the *x*-axis, with the annotated genomic organization of SVA displayed at the bottom for reference.

To investigate whether recombination contributes to the genetic diversification of SVA, we first assessed genome-wide recombination signals using SplitsTree. Network analysis revealed extensive reticulation among SVA genomes, indicating a strong overall recombination signal across the dataset (Figure 2A). This result supports the widespread occurrence of recombination during SVA evolution, without assigning specific events to particular time periods or geographic regions.

We next sought to identify specific recombinant viruses using RDP4. By applying seven independent detection methods and requiring statistically significant support from at least four algorithms (*p* < 0.05), we identified eight high-confidence recombinant strains (Table 1). All recombinant viruses were sampled in China between 2017 and 2019 and clustered within epidemic-associated lineages, consistent with the timing of large-scale SVA outbreaks and the emergence of novel variants. Parental lineage analysis further revealed that all detected recombination events occurred within lineage 2, consistent with intralineage recombination and suggesting relatively active genetic exchange within this lineage.

Recombination breakpoints were non-randomly distributed across the genome, with a predominant concentration in the 2C region (Figure 2B). The 2C protein is a conserved nonstructural protein with ATPase activity and is thought to play important roles in viral RNA replication, replication-complex assembly, and membrane remodeling. Together, these findings suggest that recombination contributes to the observed genetic diversity of SVA during recent outbreaks, as evidenced by the detection of multiple recombinant strains and intralineage recombination patterns. These results indicate the occurrence of genetic exchange among co-circulating viruses under epidemic conditions.

### 3.3. Haplotype Network Reveals Global Dissemination and Regional Diversification of SVA

**Figure 3:**
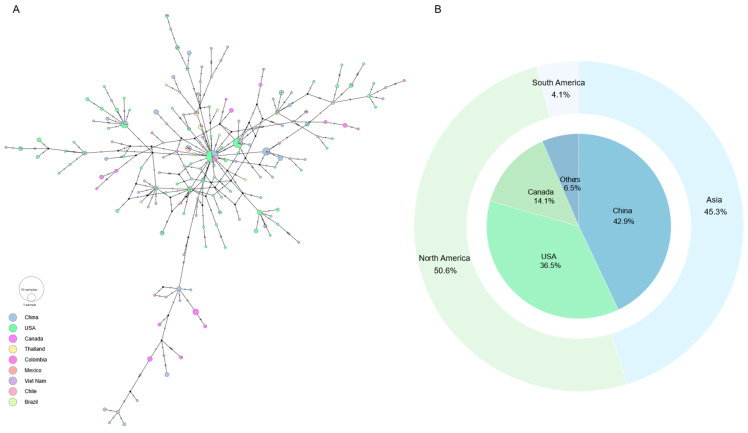
Haplotype network and geographic distribution of the SVA 3AB gene. (**A**) Haplotype network of the SVA 3AB gene colored by country. Each node represents a distinct haplotype, with node size proportional to the number of sequences sharing that haplotype. Connections between nodes represent mutational steps, and node colors indicate the country of origin. (**B**) Global and country-level distribution of SVA 3AB haplotypes. The outer circular chart represents the distribution of haplotypes across different continents, with colors indicating different continents. The inner pie chart shows the distribution of haplotypes among different countries, with colors corresponding to different countries.

To further characterize the evolutionary dynamics and geographic dissemination of SVA, we performed haplotype analysis based on nucleotide sequences of the nonstructural gene 3AB. A total of 329 sequences yielded 170 distinct haplotypes (Figure 3A), reflecting substantial genetic diversity within this region.

The central haplotype was the most prevalent and included strains sampled from China, the United States, Canada, and Brazil, demonstrating broad geographic distribution. In contrast, most other haplotypes were represented by few sequences and were geographically restricted. When stratified by sampling location, 42.9%, 36.5%, and 14.1% of haplotypes were associated with strains collected in China, the United States, and Canada, respectively (Figure 3B).

The haplotype network exhibited a structured topology characterized by a high-frequency central node connected to numerous low-frequency derivatives. This star-like backbone is consistent with recent expansion of a dominant ancestral variant. Importantly, several peripheral haplotypes formed secondary branches rather than remaining terminal, generating localized subclusters within the network. Such hierarchical branching indicates that, following global dissemination of major variants, continued mutation accumulation and regional transmission contributed to ongoing diversification.

### 3.4. Pervasive Purifying Selection Constrains the Evolution of SVA

To evaluate the role of natural selection in shaping SVA evolution, we conducted selection pressure analyses using 18 representative strains sampled from major phylogenetic clades and geographic regions. Codon-based maximum likelihood analyses implemented in PAML revealed that the overall *ω* (dN/dS) values for multiple genes, including 2AB, 2C, 3C, 3D, and VP1–VP3, were consistently below 1 (Table 2). These results indicate that SVA coding sequences are predominantly subject to purifying selection.

**Figure 4:**
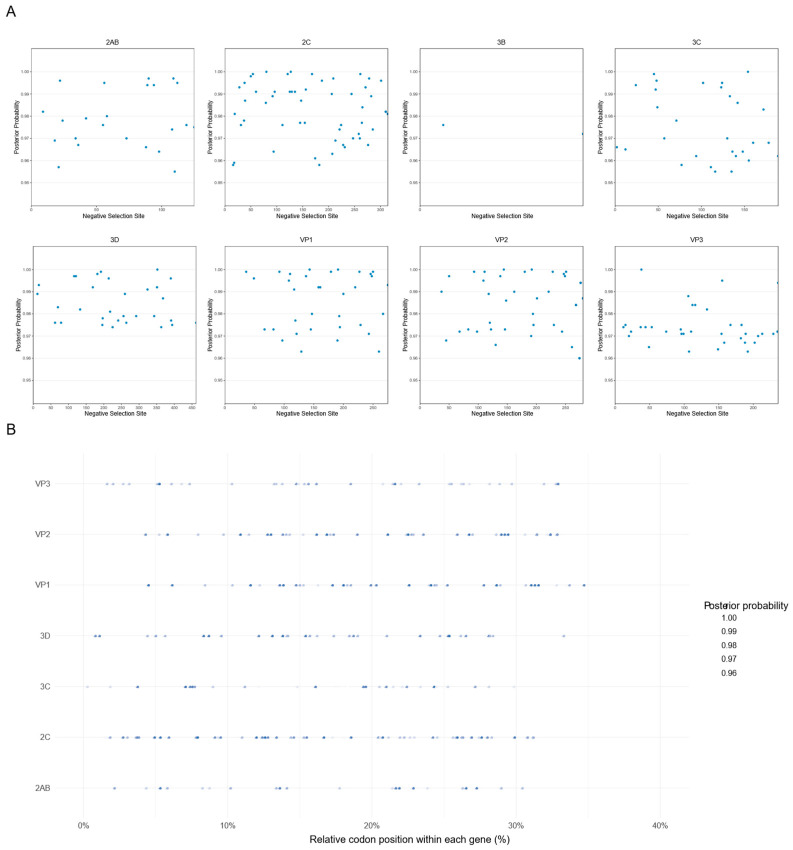
Purifying selection across SVA genes. (**A**) Scatter plot of negatively selected sites in SVA genes. In each plot, the *y*-axis represents the posterior probability that a site is under negative selection (dN/dS < 1), as inferred from sites consistently detected by three complementary methods (MEME, FEL, and FUBAR), and the *x*-axis represents the positions of negatively selected sites within each gene. (**B**) Relative positions of negatively selected sites across SVA genes. The *x*-axis represents the relative positions of negatively selected sites, and the *y*-axis corresponds to gene names. The color intensity of each point reflects the magnitude of the posterior probability.

To detect potential episodic or lineage-specific positive selection, additional analyses were performed using MEME, FEL, and FUBAR models available on the Datamonkey platform. The MEME model identified two codon sites under statistically significant episodic positive selection in the 3D gene (*p* < 0.05) (Table 3). In contrast, neither FEL nor FUBAR detected pervasive positive selection across genes, but both methods identified numerous codons evolving under significant negative selection, consistent with the low genome-wide *ω* values (Figure 4A).

Mapping negatively selected sites across coding regions revealed a non-uniform distribution, with a higher density observed in the 5′ portions of several genes, corresponding to N-terminal protein domains (Figure 4B). This pattern indicates heterogeneous functional constraint across the genome, with certain regions exhibiting stronger evolutionary conservation. Collectively, these results demonstrate that purifying selection is widespread across the SVA genome, while only limited evidence of episodic positive selection was detected.

### 3.5. Temporal Dynamics and Evolutionary Expansion of SVA

**Figure 5:**
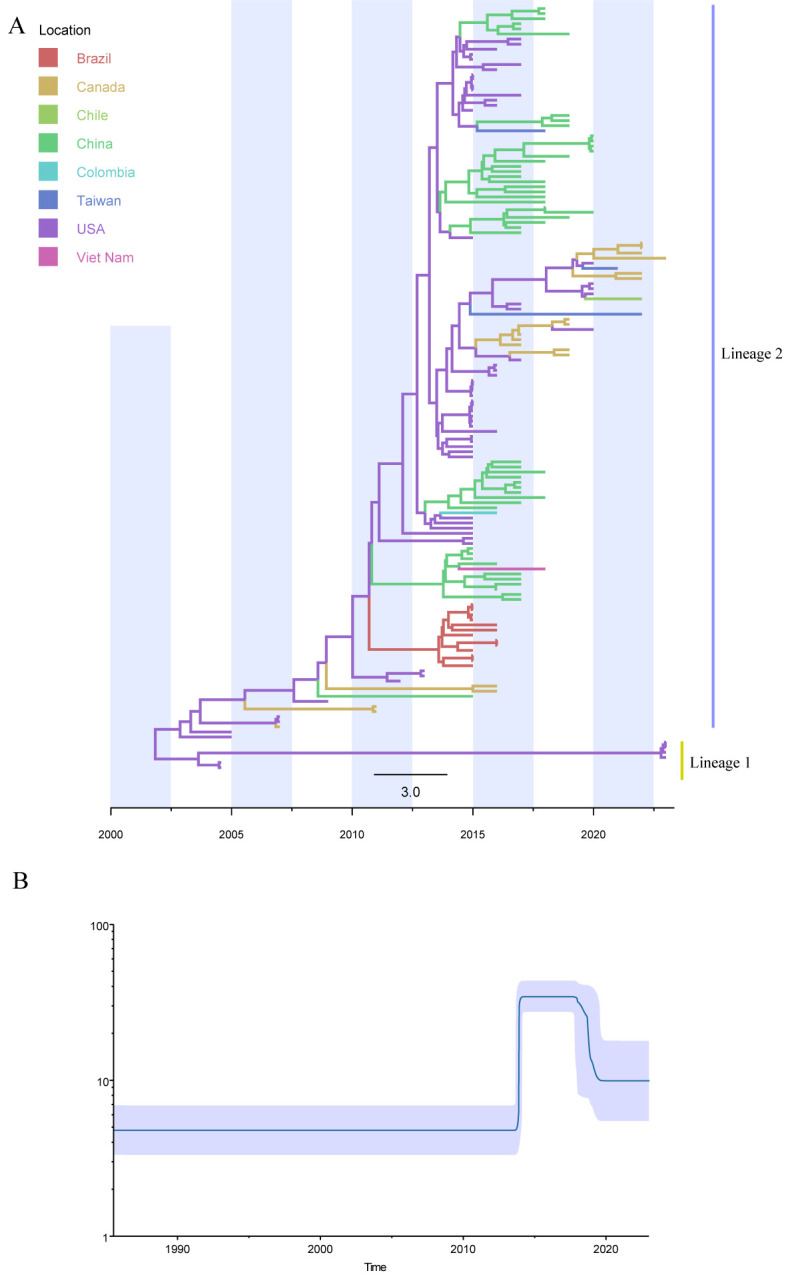
Time-scaled phylogeny and effective population size dynamics of SVA. (**A**) Time-scaled phylogeny of SVA. Branch lengths are proportional to time, with the *x*-axis representing years. The tree highlights two major SVA lineages, and branch colors correspond to the countries of origin. (**B**) Bayesian skyline plot of SVA. Bayesian skyline analysis illustrating temporal changes in the effective population size of SVA. The *y*-axis represents the effective population size (Ne), and the *x*-axis represents time (years).

To investigate the temporal dynamics of SVA evolution, we constructed a time-scaled phylogeny using BEAST based on the previously inferred phylogenetic structure (Figure 5A). The resulting tree recapitulated the distinction between Lineage 1, dominated by historical strains from the United States, and Lineage 2, which has circulated across multiple regions. Around 2012, an increase in branching density became apparent, indicating concurrent diversification within the SVA population, which was largely driven by lineage 2 strains from China and the United States.

Bayesian Skyline analysis revealed that the effective population size remained low and stable prior to 2010, followed by a pronounced exponential increase beginning around 2014 (Figure 5B). This demographic expansion aligns with the lineage diversification observed in the time-scaled phylogeny, suggesting that SVA underwent a phase of rapid evolutionary expansion during the early 2010s.

### 3.6. Phylogeographic Patterns of SVA with Emphasis on East Asia

**Figure 6:**
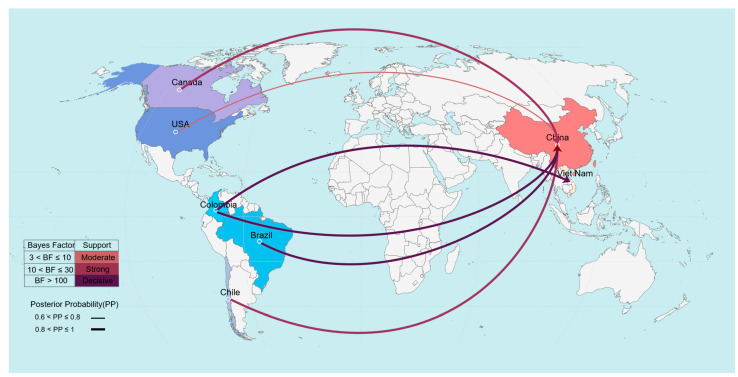
Phylogeographic reconstruction of SVA dissemination. Arrows indicate the direction of viral spread between countries or regions, with arrow thickness proportional to the posterior probability of the inferred transmission routes and arrow color reflecting the strength of support (Bayes factor).

To examine the global dissemination of SVA, we performed a discrete phylogeographic analysis using BEAST (Figure 6). Migration pathways supported by Bayes factors > 3 and posterior probabilities > 0.5 were retained for interpretation. The analysis indicated extensive viral exchanges between the Americas and Asia, with asymmetry in cross-regional diffusion. The most strongly supported routes (BF > 100; PP > 0.8) corresponded to viral movements from countries in the Americas to East Asia. Strains from East Asia were frequently inferred as recipients of SVA lineages from multiple source countries. In addition, transmission from Colombia to Thailand was supported by similarly high Bayes factor and posterior probability.

These results highlight heterogeneous geographic spread of SVA and suggest that East Asia has been a prominent recipient region in the recent dissemination of viral lineages, although the precise contribution to global transmission should be interpreted in the context of sampling limitations.

## 4. Discussion

Over the past two decades, SVA has emerged as a globally relevant swine pathogen, with outbreaks increasingly reported across multiple continents [1]. Its rapid spread, combined with clinical presentations resembling notifiable vesicular diseases such as Foot-and-Mouth Disease [35], underscores the challenges of timely detection and effective control. Our comprehensive analyses of publicly available SVA genomes reveal that viral evolution is shaped by a combination of frequent mutation, intra-lineage recombination, and strong purifying selection, resulting in high genetic diversity while maintaining functional constraints on key proteins [36,37,38]. These findings highlight a dynamic evolutionary landscape in which a limited number of successful lineages have driven global dissemination, yet localized diversification continues to generate novel variants. These observations are also consistent with the broader possibility of historical and ongoing interspecies transmission events in swine, which may have contributed to viral establishment and diversification.

Together, this study provides an integrated perspective on the mechanisms underpinning the emergence and spread of SVA, with implications for molecular surveillance, vaccine design, and risk assessment. Specifically, the identified phylogenetic clustering and geographic structure highlight the importance of lineage-based genomic surveillance for tracking viral spread. The purifying selection across the genome suggests relative conservation of most viral proteins, supporting the feasibility of targeting conserved regions for vaccine development, while the presence of limited within-lineage recombination indicates that caution should be taken when selecting genomic regions for molecular epidemiological markers. In addition, the time-scaled phylogenetic and phylogeographic analyses provide insights into the temporal dynamics and spatial dissemination of SVA, which may inform risk assessment and outbreak monitoring strategies.

Phylogenetic reconstruction revealed a clear separation of SVA into two major evolutionary lineages with distinct temporal and geographic distributions. Lineage 1 primarily comprises early strains circulating in the United States before 2007, whereas Lineage 2 includes the majority of more recent isolates that have spread across multiple regions. This pattern suggests that contemporary SVA diversity is largely derived from a globally disseminated lineage that emerged after the mid-2000s [37]. The predominance of sequences from China and the United States likely reflects both the epidemiological importance of SVA in these countries and differences in surveillance intensity and sequencing capacity [39]. Nevertheless, the strong temporal structure observed in the phylogeny indicates that the emergence and subsequent global spread of Lineage 2 represent a key transition in the evolutionary history of SVA.

Recombination analysis further indicates that genetic exchange has contributed to the diversification of contemporary SVA populations. All detected recombination events occurred within Lineage 2 and were associated with strains sampled during recent outbreak periods, suggesting that recombination may be facilitated under conditions of high viral prevalence and co-circulation of related variants. Recombination breakpoints were predominantly located within nonstructural regions, particularly the 2C gene. The 2C protein plays a central role in viral replication and replication-complex formation in many picornaviruses [40,41], and recombination occurring in this region may allow the reassortment of functional modules involved in replication efficiency and host adaptation. Although the precise functional consequences of these events remain unclear, the observed recombination patterns highlight the potential role of genetic exchange in accelerating the emergence of novel genomic configurations during epidemic transmission [42,43].

Haplotype analysis of the 3AB gene revealed substantial genetic diversity, with 170 distinct haplotypes identified among 329 sequences. This high haplotype richness indicates that SVA undergoes frequent mutation, generating a wide array of genetic variants [44]. The haplotype network exhibited a star-like topology, in which a central, high-frequency haplotype was connected to numerous low-frequency derivatives. Such a structure is typically associated with recent population expansion from a successful ancestral variant. The presence of a globally distributed central haplotype spanning multiple countries suggests that a limited number of lineages have contributed disproportionately to current SVA diversity.

In addition to the central star, several peripheral haplotypes formed secondary branches and localized subclusters, indicating that ongoing mutation accumulation and regional transmission have further diversified the viral population following its global dissemination. Focusing on the 3AB gene provides a manageable framework to resolve haplotype diversity while still capturing these patterns, as full-genome haplotype analysis would generate even greater complexity. Nonetheless, even within this single gene, the combination of extensive haplotype richness, hierarchical branching, and geographic differentiation underscores the virus’s potential for rapid evolution and adaptive diversification. Overall, these patterns of high mutational input, global dissemination, and regional differentiation are consistent with the epidemiological history of SVA and with patterns observed in other emerging RNA viruses.

Despite the accumulation of substantial genetic variation, selection pressure analyses indicate that the SVA genome remains predominantly constrained by purifying selection. Genome-wide dN/dS ratios were consistently below one across multiple genes, and only limited evidence of episodic positive selection was detected. Notably, unlike many emerging RNA viruses that frequently exhibit adaptive evolution driven by host immune pressure or ecological shifts, SVA appears to experience pervasive purifying selection across much of its genome. In particular, negatively selected sites were concentrated in the N-terminal core domains of several structural and nonstructural proteins, suggesting that these regions are subject to strong structural and functional constraints. Such constraints likely reflect the essential roles of viral proteins involved in capsid assembly, RNA replication, and replication-complex formation, where even minor amino acid changes may compromise viral fitness [45].

At the same time, the coexistence of extensive haplotype diversity and predominantly purifying selection suggests an evolutionary pattern characterized by high mutational input coupled with strong selective filtering. Although mutations arise continuously during viral replication, most appear to be selectively removed from the population, resulting in the retention of functionally constrained variants. This dynamic may explain why substantial nucleotide-level diversity can accumulate without corresponding levels of adaptive amino acid change. Consequently, SVA evolution may be characterized by rapid generation of genetic variants followed by strong purifying filtering that preserves the functional integrity of key viral proteins. Such evolutionary dynamics may explain why major antigenic and functional regions remain relatively conserved despite ongoing accumulation of genetic diversity at the nucleotide level.

Temporal reconstruction of SVA evolution revealed additional insights into the demographic history of the virus. Time-scaled phylogenetic analysis indicated a noticeable increase in lineage diversification around the early 2010s, followed by a rapid rise in effective population size beginning around 2014 [46]. These findings are consistent with epidemiological reports describing the emergence of widespread SVA outbreaks during this period. Together with the star-like haplotype network structure, these results suggest that the current diversity of SVA largely reflects a relatively recent phase of rapid population expansion and lineage turnover, rather than long-term accumulation of deeply diverged lineages.

Spatial diffusion analysis further highlights the global dimension of SVA transmission. Phylogeographic reconstruction revealed frequent viral exchanges between the Americas and Asia, with strongly supported migration routes indicating repeated introductions from the Americas into East Asia. This asymmetric pattern of cross-regional diffusion likely reflects the structure of international swine production and trade networks, which can facilitate long-distance movement of pathogens. Furthermore, the presence of additional inferred transmission pathways not readily explained by direct trade relationships suggests that other mechanisms, such as movement of biological products, feed ingredients, or indirect human-mediated transport, may also contribute to the spread of SVA across borders.

Several limitations should be considered when interpreting these findings. First, the available genomic dataset is unevenly distributed geographically and temporally, with a large proportion of sequences originating from a limited number of countries and recent time periods. Such sampling heterogeneity may bias discrete phylogeographic inference by overestimating transition rates involving heavily sampled locations, and may also influence demographic reconstruction, as the increase in effective population size coincides with a substantial rise in sequence sampling. Approaches such as structured coalescent-based models and more balanced temporal sampling may help mitigate these effects in future studies. Second, although recombination was detected in a small fraction of sequences and was confined within major phylogenetic lineages, such within-lineage recombination may still introduce minor uncertainty in time-scaled phylogenetic inference. Finally, the reliance on publicly available sequences may underestimate the true diversity and frequency of recombination events occurring in natural populations. Expanded genomic surveillance in underrepresented regions will therefore be essential to refine our understanding of SVA evolutionary dynamics.

In summary, this study reveals a dynamic evolutionary landscape in which SVA maintains core functional constraints while generating genetic diversity through mutation and recombination within a rapidly expanding global lineage. The combination of recent population expansion, ongoing recombination, and increasing transcontinental transmission underscores the importance of sustained genomic surveillance and international cooperation in monitoring the future spread of this emerging swine pathogen. These evolutionary processes suggest that continued viral diversification or the emergence of related variants through recombination or introduction from external reservoirs remains a potential risk.

## 5. Conclusions

This study demonstrates that SVA evolution is characterized by the interplay of frequent mutation, intra-lineage recombination, and pervasive purifying selection, resulting in substantial genetic diversity while preserving essential viral functions. The emergence and global dissemination of a dominant lineage after the mid-2000s, together with rapid population expansion in the early 2010s, have shaped the current epidemiological landscape of SVA. Recombination, particularly within nonstructural regions, may further accelerate viral diversification during epidemic transmission. Meanwhile, phylogeographic patterns indicate repeated interregional spread, likely driven by human-mediated activities such as international swine trade. Despite these advances, uneven genomic sampling remains a limitation, underscoring the need for broader surveillance. Overall, these findings highlight the importance of continuous genomic monitoring and coordinated international efforts to better understand, predict, and control the ongoing spread of SVA.

## Figures and Tables

**Table 1:** Collection years and locations of recombinant strains.

Accession No.	Collection Year	Location
MH316113.1	2017	China
MF189001.1	2017	China
MK357117.1	2018	China
MK357116.1	2018	China
MG765559.1	2017	China
MN887249.1	2019	China
MK284515.1	2018	China
MW713120.1	2018	China

**Table 2:** Selection pressure analysis based on PAML (*ω* values).

Gene	*ω*
2AB	0.0379
2C	0.0283
3B	0.2248
3C	0.0297
3D	0.0596
L	0.0581
VP1	0.0243
VP2	0.0243
VP3	0.0414
VP4	0.0179

**Table 3:** Selection pressure analysis results based on Datamonkey.

Gene	MEME (*p*-Value)	FUBAR (Posterior Probability)	FEL (*p*-Value)
3D	122 (0.020)	–	–
3D	137 (0.043)	–	–

## Data Availability

The original contributions presented in this study are included in the article. Further inquiries can be directed to the corresponding authors.

## Abbreviations

The following abbreviations are used in this manuscript:

RNA	Ribonucleic Acid
DNA	Deoxyribonucleic Acid
MAFFT	Multiple Alignment using Fast Fourier Transform
BIC	Bayesian Information Criterion
RDP4	Recombination Detection Program version 4
FEL	Fixed Effects Likelihood
MEME	Mixed Effects Model of Evolution
FUBAR	Fast Unconstrained Bayesian AppRoximation
MCMC	Markov Chain Monte Carlo
ESS	Effective Sample Size
BSSVS	Bayesian Stochastic Search Variable Selection
